# Odors enhance the salience of matching images during the attentional blink

**DOI:** 10.3389/fnint.2013.00077

**Published:** 2013-11-06

**Authors:** Amanda K. Robinson, Jason B. Mattingley, Judith Reinhard

**Affiliations:** ^1^Queensland Brain Institute, The University of QueenslandBrisbane, QLD, Australia; ^2^The School of Psychology, The University of QueenslandBrisbane, QLD, Australia

**Keywords:** cross-modal perception, multisensory integration, olfaction, visual attention, attentional blink

## Abstract

As any food critic knows, the visual presentation of a dish can enhance its aroma. Is the reverse also true? Here we investigated whether odors can enhance the salience of familiar visual objects at the limits of perceptual discrimination, using rapid serial visual presentations (RSVP) to induce an attentional blink (AB). We had participants view RSVP streams containing photographs of odor-related objects (lemon, orange, rose, and mint) amongst non-odor related distractors. In each trial, participants inhaled a single odor, which either matched the odor-related target within the stream (congruent trials), did not match the odor-related target (incongruent trials), or was irrelevant with respect to the target. Congruent odors significantly attenuated the AB for odor-related visual targets, compared with incongruent and irrelevant odors. The findings suggest that familiar odors can render matching visual objects more salient, thereby enhancing their competitive strength at the limits of temporal attention.

## INTRODUCTION

Odors are variable mixtures of molecules that bind preferentially to specialized odor receptors in the nasal epithelium. Odors are categorized in a similar manner to familiar visual stimuli, as unified “object” percepts rather than as a combination of individual parts (Gottfried, 2010). From an early age we learn to associate odors with their sources in the environment (Engen, 1987; Hvastja and Zanuttini, 1989). Indeed, the first-learned association between an odor and a visual object is more strongly encoded in long-term memory than subsequent odor-object pairings, and is associated with unique patterns of brain activity in the left hippocampus (Yeshurun et al., 2009). The well-learned associations between object features such as color, shape, odor, and texture provide a potential foundation for multi-sensory interactions (Walla, 2008). From a biological perspective, the binding of olfactory information with other sensory and semantic features is particularly interesting because unlike vision, audition and touch, olfactory inputs reach the olfactory cortex without first passing through the thalamus (Smythies, 1997). The locus of any interactions between olfaction and other sensory modalities therefore is likely to arise in higher cortical areas (Spence et al., 2001).

It is widely believed that odor perception is dominated by vision (Thesen et al., 2004). Visual features such as color and shape can influence olfactory detection (Gottfried and Dolan, 2003), odor preference and intensity judgments (Sakai et al., 2005), and odor identification (Dematte et al., 2009). By contrast, little work has been devoted to the question of whether olfaction can influence visual perception, though the relevant literature does contain some tantalizing clues. When inhaling a familiar odor, for example, people tend to fixate longer, and more often, on a visual image that matches the odor than when no odor is present (Seo et al., 2010). People also make eye movements toward visual stimuli faster when smelling a matching odor compared with a non-matching odor (Seigneuric et al., 2010). Moreover, psychophysiological studies using event-related potentials (ERPs) have shown that the N400 component is enhanced when participants are presented with a visual stimulus immediately after a matching (congruent) olfactory prime, relative to the same visual stimulus preceded by a mismatching (incongruent) odor (Grigor et al., 1999; Sarfarazi et al., 1999; Castle et al., 2000). Taken together, these studies suggest that olfaction can be used to guide visual search and other aspects of visual behavior that are under voluntary control.

Recent studies have also suggested that olfaction can influence relatively early, non-voluntary, aspects of visual perception. Zhou et al. (2010) found that visual images dominated for longer during binocular rivalry when participants inhaled matching odors than when they inhaled mismatching odors, and that this effect disappeared when participants inhaled only water vapor but were told the odor matched one of the visual images. This congruency effect was more pronounced when matching olfactory and visual stimuli were processed by the same brain hemisphere (Zhou et al., 2012).

Taken together, the findings from previous investigations of olfactory-visual interactions suggest that visual images become more perceptually salient if they are presented in the context of a matching odor. Based on this evidence, we predicted that matching odor-image pairs should exhibit enhanced competitive strength under conditions in which multiple visual stimuli are presented in rapid succession. To test this prediction, we paired matching and non-matching odors with target pictures embedded within a rapid serial visual presentation (RSVP) stream, and measured the depth of the attentional blink (AB) for the visual targets. In such tasks, the second of two visual targets (T2) in the RSVP stream is impaired if it is presented within 200–500 ms of the first target (T1) (Raymond et al., 1992), reflecting a bottleneck in the temporal allocation of selective attention. The paradigm has been used to determine the salience of particular stimuli by manipulating the visual properties of T1 and T2. Importantly, increasing the visual salience of T2 (e.g., using emotionally arousing pictures or words) has been found to attenuate the AB (Keil and Ihssen, 2004; De Martino et al., 2009).

By pairing a matching odor with a visual T2 (e.g., a lemon scent inhaled during a trial that could contain a picture of a lemon), we tested whether odors enhance the salience of a matching visual image and thus increase the likelihood of it being identified within an RSVP stream. We also conducted a control experiment using word cues (e.g., the word “lemon”) rather than odors, to determine whether the mere concept of an odor influences T2 discrimination, independent of any effects of the odors themselves.

## MATERIALS AND METHODS

### PARTICIPANTS

This study was approved by the human ethics committee of The University of Queensland in accordance with the National Health and Medical Research Council’s guidelines. Informed consent was obtained from all subjects. Twenty participants (11 females, 9 males; age range 19–35 years) for Experiment 1 and nineteen participants (13 females, 6 males; age range 20–26 years) for Experiment 2 were recruited from The University of Queensland. Participants were screened for their ability to distinguish between the test odors, and completed a questionnaire relating to factors such as age, gender, odor allergies and whether they were taking hormone-related medications. All of the participants who took part in the experiments reported normal olfactory perception, normal or corrected-to-normal vision, were non-smokers and had no known odor allergies.

### STIMULI

**FIGURE 1:**
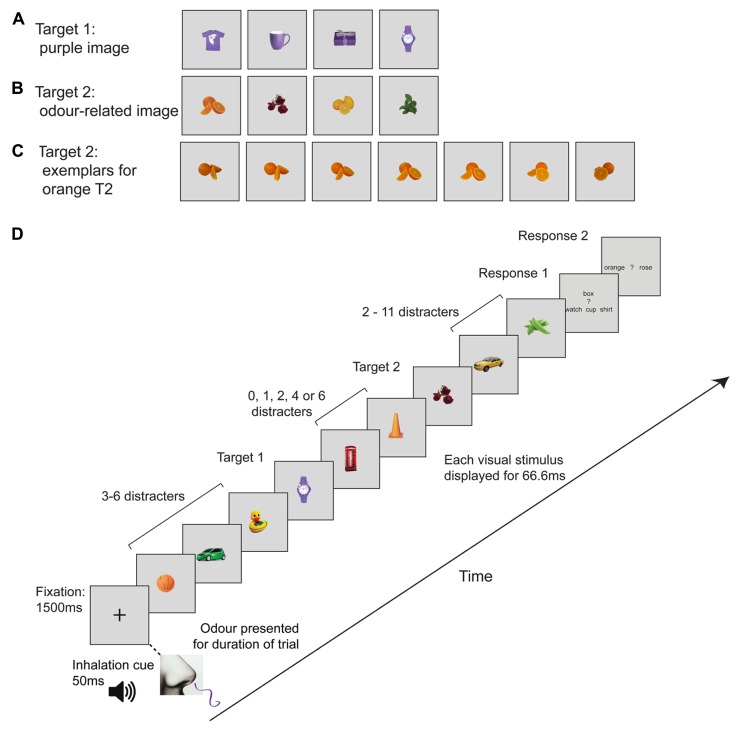
**Schematic representation of the rapid serial visual presentation (RSVP) trial sequence and visual images.** **(A)** Target 1 (T1) images (purple object; watch, mug, *t*-shirt, or box). **(B)** Target 2 (T2) images; orange, rose, lemon or mint **(C)** Example images for Orange T2. There were seven exemplars of each T2, comprising photos of the same object taken from different angles. **(D)** Example RSVP sequence. The fixation cross was presented for 1500 ms and then 16 images appeared one after another at 67 ms each. T1 and T2 were separated by 0, 1, 2, 4, or 6 images, which corresponds to T1–T2 lags of 1, 2, 3, 5, or 7. An odor presented for the entire duration of each trial was either congruent, incongruent or irrelevant. Trials involving plain air (“no odor”) were randomly intermingled as a means of verifying the presence of an AB in the absence of olfactory stimulation.

Both experiments employed two tasks (single or dual target detection), three cue conditions (incongruent, congruent, irrelevant), and five lags (T2 followed T1 by 1, 2, 3, 5, or 7 pictures). We also included a visual-only baseline, in which participants inhaled plain air, to establish that the images used elicited a characteristic AB effect. The first target, T1, was randomly selected from a set of four purple images (*t*-shirt, mug, box, or watch; see **Figure 1A**). The second target, T2, was an image of roses, mint leaves, orange, or lemon (see **Figure 1B**). There were seven exemplars of each target object (see **Figure 1C**). Participants only had to distinguish between two possible T2 objects on any given trial. The combination of T2 images was always the same; on half the trials, T2 was rose or orange, while on the other half of trials T2 could be lemon or mint. The non-target (distractor) images were randomly selected from a set of 20 pictures of ordinary objects that do not have a typical or “canonical” odor (e.g., hat, tree, basketball). These were the same size as the other images, and each distractor was the same color as one of the critical targets (i.e., red, green, yellow, or orange). The Cogent Toolbox in Matlab was used to present the visual stimuli on a 27-inch LCD monitor. Participants placed their chin on a chin rest approximately 60 cm from the monitor.

In Experiment 1, the single odor presented for each trial was congruent, incongruent or irrelevant with respect to the critical target object (T2). The odors lemon, orange, mint, and rose (Queen Fine Foods Ltd., Flavouring Essences) were used as congruent and incongruent odors, and coffee essence (Queen Fine Foods Ltd., Flavouring Essence) was used as an irrelevant odor. A four-channel olfactometer delivered odors to participants by driving clean air at 0.6 L per minute through vials containing drops of undiluted odor solution. On one quarter of trials, clean air was presented as a “no odor” condition. The “no odor” trials were intermingled with the critical odor trials. Odors from the four lines emerged at a funnel placed in front of the chin rest, under the participants’ nose.

There were four blocks in the experiment, one for each combination of task (single or dual target detection) and stimulus combination (rose/orange or lemon/mint). In each block, there were 160 trials, with each combination of lag, task, and odor condition repeated four times. Importantly, within each block the targets were paired an equal number of times with each odor, so an odor was never predictive of the visual target. Trials were randomized within each block, with the exception that an odor was never repeated more than three times in a row, to reduce the chance of olfactory habituation. Block order was counterbalanced between participants.

### PROCEDURE

On each trial, participants had to inhale an odor and then detect one target (single task) or two targets (dual task) within an RSVP stream containing images of real objects (see **Figure 1**). At the start of each trial, a 400-Hz sine wave tone was presented to participants for 50 ms as a cue to inhale through their nose, to ensure they perceived the odor that was delivered. A central fixation cross appeared at the same time as the auditory cue. The cross remained on screen for 1000 ms and was followed by 16 images shown sequentially at 15-Hz (i.e., 67 ms per item) with no gap between successive pictures. Odors were delivered from the onset of visual fixation until the end of the trial (i.e., for approximately 2 s). At the end of the trial, participants had to indicate which target or targets had been displayed during the trial. In the single-task version of the protocol, the monitor displayed the names of the T2 target objects that might have been displayed during the trial, for example “lemon or mint?” Participants reported which of the two objects had been present by pressing the left or right arrow keys on a standard keyboard. In the dual task version, the monitor first displayed the names of the T1 objects (“shirt box watch cup”) and participants indicated the identity of the T1 object that was displayed using a button press to the up, down, left, or right arrow keys. Following the T1 response, participants indicated their response for the T2 object using another button press. No feedback was given regarding accuracy.

We anticipated that participants’ visual performance might be influenced by the name of each odor and its associated conceptual representation. We therefore conducted Experiment 2 with a new group of participants to investigate the effect of matching and non-matching word cues on visual target discrimination, without any corresponding odors. The experiment involved an identical RSVP task but with a single-word cue presented at the start of the stream rather than an odor. The word was either congruent or incongruent with the T2 object (“lemon,” “mint,” “orange,” “rose”), or was irrelevant (“coffee”) with respect to the T2 object. A trial consisted of the fixation cross for 500 ms, the word cue for 500 ms, the fixation cross again for 500 ms, followed by the 16 visual images presented at 15 Hz. To keep the procedure as comparable to Experiment 1 as possible, plain air was delivered using an olfactometer and participants were cued to inhale through their nose at the start of each trial.

### DATA ANALYSIS

T2 accuracy in the dual task was calculated as a proportion of trials in which T1 was correctly identified (T2|T1). As participants only had to distinguish between two T2 objects, chance performance was 50%. For each experiment, a 2 × 3 repeated measures analysis of variance (ANOVA) was conducted with factors of lag and cueing condition (congruent, incongruent, and irrelevant). Follow-up pairwise analyzes were performed to compare T2|T1 accuracy at the lag yielding the lowest performance (i.e., the deepest point of the AB) with accuracy at lag 7 (Bowman and Wyble, 2007) for each cue condition. To compare between Experiments 1 and 2, an independent samples *t*-test was conducted for each of the critical conditions (congruent, incongruent, and irrelevant) to determine the effect of cue type (odor versus word cue) on T2|T1 accuracy. An alpha level of 0.05 was used for all statistical tests.

## RESULTS

**FIGURE 2:**
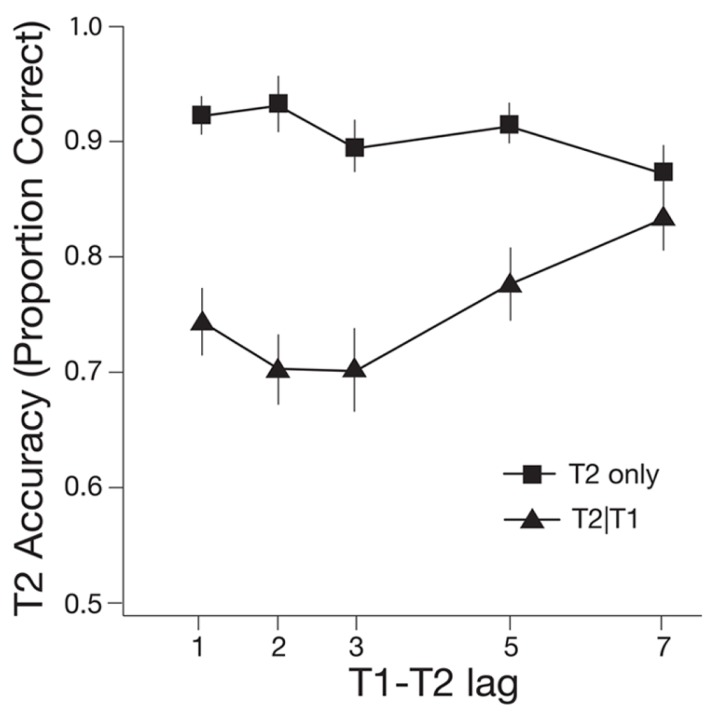
**Mean accuracy of Target 2 (T2) detection for single-task (T2 only) and dual task (T2|T1) plotted as a function of T1–T2 lag for the baseline (no odor) condition.** One lag corresponds with 66.6 ms. Error bars represent one standard error of the mean.

Experiment 1 was designed to determine if an odor cue modulated performance in an RSVP task involving matching versus non-matching targets. The baseline (no odor) condition resulted in a typical AB effect (see **Figure 2**). In the dual task, T1 was correctly identified on 86.25% of trials and this did not vary across lag. A one-way repeated measures ANOVA revealed that T2|T1 accuracy varied significantly across lag, *F*(4,76) = 4.61, *p* = 0.002, $\eta_{p}^{2}$ = 0.20. Simple effects tests revealed accuracy at lag 7 was significantly higher than accuracy at lag 2, *t*(19) = 3.59, *p* = 0.002, and lag 3, *t*(19) = 4.35, *p* < 0.001.

**FIGURE 3:**
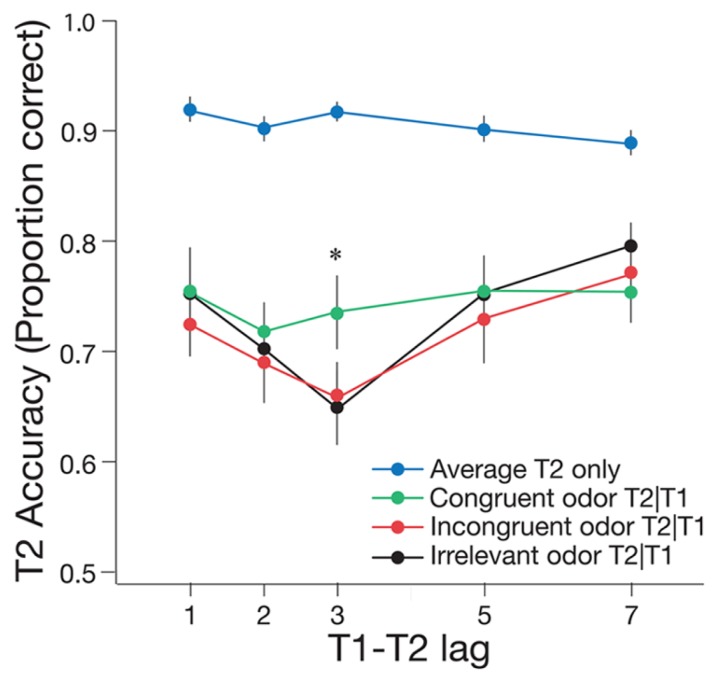
**Mean accuracy of T2|T1 identification for the congruent, incongruent, and irrelevant odor conditions of Experiment 1, plotted as a function of T1–T2 lag.** Mean accuracy for identifying T2 only is also shown, pooled across the three odor conditions. One lag corresponds with 66.6 ms. Error bars represent one standard error of the mean. *p < 0.05.

For the critical odor conditions, typical AB effects were evident in the incongruent and irrelevant odor conditions, but the AB was attenuated in the congruent odor condition (see **Figure 3**). A 2 × 3 repeated measures ANOVA was conducted to determine whether T2|T1 accuracy varied across lag (during the AB at lag 3 and after the AB at lag 7) and odor cue (congruent, incongruent, and irrelevant). This analysis revealed a significant main effect of lag, *F*(1,19) = 20.48, *p* < 0.001, $\eta_{p}^{2}$ = 0.52, such that T2|T1 accuracy was lower at lag 3 than at lag 7. There was no significant main effect of condition, *F*(2,38) = 0.82, *p* = 0.447, $\eta_{p}^{2}$ = 0.01, but there was a marginally significant interaction between lag and odor cue, *F*(2,38) = 3.11, *p* = 0.056, $\eta_{p}^{2}$ = 0.14. Follow-up paired *t*-tests using the Bonferroni correction (α = 0.017) revealed performance at lag 3 was significantly lower than at lag 7 with an irrelevant odor, *t*(19) = -3.44, *p* = 0.003, and with an incongruent odor, *t*(19) = -3.20, *p* = 0.005. Conversely, there was no significant difference in T2|T1 accuracy between lag 3 and lag 7 in the congruent condition, *t*(19) = -0.53, *p* = 0.600. Taken together, these results suggest that inhalation of a matching odor attenuated the AB for visual stimuli.

The effect of odor condition was further analyzed by calculating the difference in T2|T1 accuracy between lag 7 and lag 3, to provide an estimate of AB magnitude per condition, and comparing this magnitude across congruent, incongruent, and irrelevant odor conditions. Pairwise *t*-tests revealed no significant difference in the magnitude of the AB between the irrelevant and incongruent odor conditions,* t*(19) = 0.60, *p* = 0.555. By contrast, the size of the AB effect was significantly smaller in the congruent condition than in the incongruent and irrelevant conditions combined, *t*(19) = -2.70, *p* = 0.012.

To summarize, the results from Experiment 1 revealed a typical AB effect for the irrelevant and incongruent odor conditions. The presence of a congruent odor, however, significantly attenuated the AB effect for the same visual stimuli.

**FIGURE 4:**
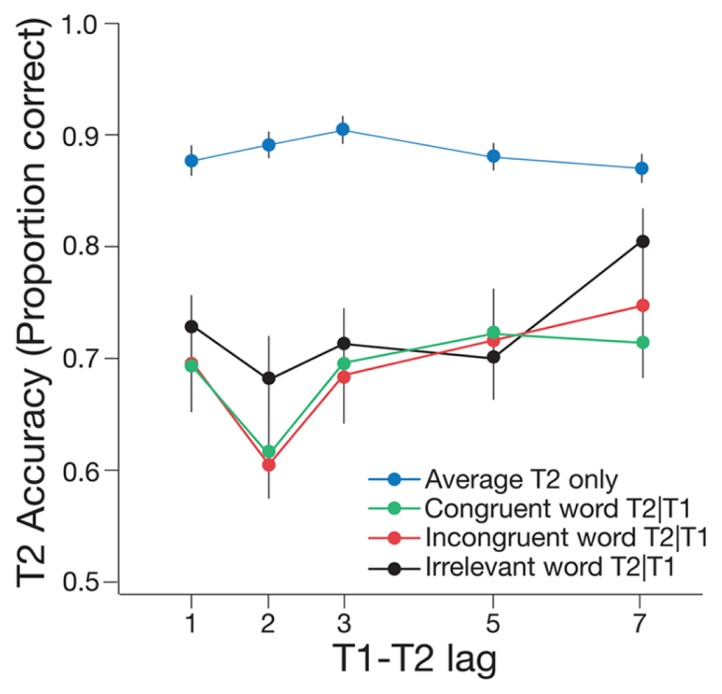
**Mean accuracy of T2|T1 identification for the congruent, incongruent, and irrelevant word conditions of Experiment 2, plotted as a function of T1–T2 lag.** Mean accuracy for identifying T2 only is also shown, pooled across the three word conditions. One lag corresponds with 66.6 ms. Error bars represent one standard error of the mean.

Experiment 2 was performed to determine whether cueing of odor-related concepts using word cues might also influence the AB, thus providing a potential explanation for the findings of Experiment 1. The factors in the experiment and the analyzes were the same as those described for Experiment 1. Typical AB effects were found for all of the critical conditions (see **Figure 4**).

A 2 × 3 repeated measures ANOVA revealed a significant main effect of lag, *F*(1,18) = 44.99, *p* < 0.001, $\eta_{p}^{2}$ = 0.71, such that T2|T1 accuracy was significantly lower at lag 2 than at lag 7. There was a significant main effect of word cue condition, *F*(2,36) = 3.85, *p* = 0.031, $\eta_{p}^{2}$ = 0.18, such that T2|T1 performance was higher in the irrelevant condition than in the congruent, *t*(37) = 3.00, *p* = 0.005, and incongruent conditions, *t*(37) = 2.59, *p* = 0.014. Importantly, there was no significant difference between the congruent and incongruent word conditions, *t*(37) = -0.44, *p* = 0.663. In addition, there was no significant interaction between lag and word cue condition, *F*(2,36) = 0.45, *p* = 0.639, $\eta_{p}^{2}$ = 0.02. Paired *t*-tests revealed that T2|T1 accuracy at lag 2 was significantly lower than at lag 7 for both the irrelevant word condition, *t*(18) = -3.77, *p* = 0.001, and the incongruent word condition, *t*(18) = -4.79, *p* < 0.001. Critically, T2|1 accuracy was also significantly lower at lag 2 than at lag 7 in the congruent condition, *t*(18) = -3.20, *p* = 0.005, indicating the presence of a reliable AB effect for matching word trials.

As in Experiment 1, the effect of word cue was further examined by calculating the difference in T2|T1 accuracy between lag 7 and lag 2, to provide an estimate of AB magnitude per condition. There was no significant difference in AB magnitude between the congruent and incongruent conditions, *t*(18) = -0.89, *p* = 0.385, the congruent and irrelevant conditions, *t*(18) = -0.52, *p* = 0.610, or the incongruent and irrelevant conditions, *t*(18) = 0.46, *p* = 0.649. To summarize, the results of Experiment 2 indicate that word cueing of odor-related concepts had no influence on the AB effect across congruent, incongruent, and irrelevant word conditions.

In a final analysis, we compared the results from Experiment 1 (odor cue) and Experiment 2 (word cue) directly. The latency of the AB effect varied between the experiments; the effect was maximal at lag 3 for odor cues in Experiment 1, and at lag 2 for word cues in Experiment 2. Importantly, however, the overall magnitude of the AB was equivalent across the two experiments. Separate, independent samples *t*-tests revealed that there was no significant difference in T2|T1 accuracy between the word cue and odor cue conditions for incongruent trials, *t*(192.56) = -1.17, *p* = 0.245, or for irrelevant trials, *t*(192.43) = -0.22, *p* = 0.823. Thus, for the incongruent and irrelevant conditions, the odor and word cue experiments produced AB effects of equal magnitude. Critically, however, T2|T1 accuracy in the congruent condition of Experiment 1, in which a matching odor was presented (*M* = 0.74), was significantly higher than T2|T1 accuracy in the congruent condition of Experiment 2 (*M* = 0.69), in which a matching word cue was presented *t*(185.57) = 2.58, *p* = 0.011.

## DISCUSSION

The aim of this study was to determine whether matching and non-matching odors can influence temporal selective attention during the AB. When participants were exposed to clean air (no odor), dual task performance was particularly low when T2 followed T1 by approximately 100 –200 ms, a characteristic AB effect (Raymond et al., 1992). Both irrelevant and incongruent odors resulted in a typical AB, yet a congruent odor showed no reliable AB effect. These results demonstrate that odors enhance the salience of matching visual targets. To our knowledge, this is the first study to show that odors can selectively facilitate performance on a visual attention task.

The baseline (no odor) condition was designed to verify that our paradigm resulted in a typical AB effect for the visual stimuli alone, rather than as a critical experimental condition. The no odor condition was not directly comparable to the odor conditions with respect to olfactory processing, so we chose to statistically compare only the congruent, incongruent and irrelevant odor conditions. For the odor conditions, the results were dependent on the relationship between the odor and T2 image. Critically, the odors and images used in the congruent and incongruent conditions were exactly the same; only the pairing between the odors and the targets was altered across conditions. Thus, the impact of odors was not generic; only the odor matching T2 enhanced its identification. Interestingly, there were no differences between the incongruent and irrelevant odor conditions, indicating that an incongruent task-related odor did not reduce performance on the visual task compared with an olfactory control. Most likely, an odor cued relevant conceptual representations linked with that odor such as color, shape and name, so that when a target appeared that matched those concepts, it was more readily identified, even during the AB. We suggest that the odor acted as a cue for a matching visual target and made the target easier to identify at the limits of temporal attention.

It might be argued that the congruency effect observed was actually a response bias toward the odor. If this were true, when participants were unsure of the T2 object shown during the RSVP stream (e.g., rose or orange) they might have responded with the identity of the odor they smelled (e.g., rose), resulting in more correct congruent trials than incongruent trials. However, participants were clearly instructed that the odor was not predictive of T2. Furthermore, we found no decrement in performance in the incongruent condition compared with the irrelevant condition, indicating that response bias was not driving the congruency effect. To further investigate the possibility of such a bias, Experiment 2 was conducted using word cues rather than odors. If there had been a response bias toward odor identity in Experiment 1, we expected that such a bias would be at least as strong with a word cue. Interestingly, we found that the maximum AB deficit occurred at lag 3 in Experiment 1 and lag 2 in Experiment 2. It could be that odor processing impacts on attentional processing in general so that the shape of the AB changes depending on whether there is a concurrent odor. It is, however, possible that the independent group of participants in each experiment showed variation for the AB. Nevertheless, results from the word cueing experiment revealed no effect of congruency on T2 performance. In fact, performance in the congruent odor condition of Experiment 1 was significantly higher than in the congruent word condition of Experiment 2. Therefore, our findings suggest that odors enhance the salience of matching visual objects in a way that words and their associated meanings do not.

The critical stimuli used in this study were common odors and pictures of their naturally occurring sources; lemon, orange, rose, and mint. The links between these odors and visual images are readily known and were designed to produce maximal olfactory-visual effects. We propose that when participants perceived an odor (e.g., rose), they were cued for concepts or features associated with that odor (e.g., the color red, the category of flowers), which allowed enhanced identification of any visual object with those features. Clearly, however, a name alone is not sufficient to increase the salience of a matching visual target; Experiment 2 showed that matching words did not enhance image discrimination. Furthermore, although connections between colors and odors are particularly strong (Dematte et al., 2006,^2009^), odor–color associations are unlikely to be responsible for the congruency effect obtained in Experiment 1, because visual distractors in the RSVP streams were chosen to share their colors with those of the target objects. If odors selectively enhanced the salience of any visual image with a matching color (e.g., all orange objects during presentation of the orange odor), then matching-colored distractor images within the stream (e.g., basketball, traffic cone; see **Figure 1D**) should also have captured attention, potentially yielding a larger AB effect in the congruent condition. Olfactory-visual integration must therefore involve strong associations between odors and object forms (e.g., orange odor and round shape) or a link between odors and a conjunction of conceptual and visual features. Our findings indicate that odors make matching visual objects more salient, thereby enhancing their identification at the temporal limits of attention.

In conclusion, we have demonstrated that the presence of odors can significantly modulate temporal visual attention depending on whether an odor is matching or non-matching with respect to a visual image. Together with previous studies showing that odors can influence binocular rivalry (Zhou et al., 2010,^2012^) and control the amount of time that people look at specific images (Seigneuric et al., 2010; Seo et al., 2010), our findings suggest that associations between odors and visual objects can alter visual salience under appropriate conditions. An important next step is to unravel the neural mechanisms that underlie the modulating effects of odors on visual perception.

## Conflict of Interest Statement

The authors declare that the research was conducted in the absence of any commercial or financial relationships that could be construed as a potential conflict of interest.
